# Quantification of left atrial strain and strain rate using Cardiovascular Magnetic Resonance myocardial feature tracking: a feasibility study

**DOI:** 10.1186/s12968-014-0060-6

**Published:** 2014-08-12

**Authors:** Johannes Tammo Kowallick, Shelby Kutty, Frank Edelmann, Amedeo Chiribiri, Adriana Villa, Michael Steinmetz, Jan Martin Sohns, Wieland Staab, Nuno Bettencourt, Christina Unterberg-Buchwald, Gerd Hasenfuß, Joachim Lotz, Andreas Schuster

**Affiliations:** 1 Institute for Diagnostic and Interventional Radiology, Georg-August-University, Göttingen, Germany; 2 Children¿s Hospital and Medical Center Joint Division of Pediatric Cardiology, University of Nebraska/Creighton University, Omaha, Nebraska; 3 Department of Cardiology and Pneumology, Georg-August-University, Göttingen, Germany; 4 Division of Imaging Sciences and Biomedical Engineering, The Rayne Institute, St. Thomas¿ Hospital, King¿s College London, London, UK; 5 Department of Pediatric Cardiology and Intensive Care Medicine, Georg-August-University, Göttingen, Germany; 6 Cardiology Department, Centro Hospitalar de Gaia/Espinho, Vila Nova de Gaia, Portugal; 7 DZHK (German Centre for Cardiovascular Research), partner site Göttingen, Göttingen, Germany

**Keywords:** Cardiovascular magnetic resonance, Feature tracking, Diastolic dysfunction, Hypertrophic cardiomyopathy, Strain, Strain rate, Atrial physiology

## Abstract

**Background:**

Cardiovascular Magnetic Resonance myocardial feature tracking (CMR-FT) is a quantitative technique tracking tissue voxel motion on standard steady-state free precession (SSFP) cine images to assess ventricular myocardial deformation. The importance of left atrial (LA) deformation assessment is increasingly recognized and can be assessed with echocardiographic speckle tracking. However atrial deformation quantification has never previously been demonstrated with CMR. We sought to determine the feasibility and reproducibility of CMR-FT for quantitative derivation of LA strain and strain rate (SR) myocardial mechanics.

**Methods:**

10 healthy volunteers, 10 patients with hypertrophic cardiomyopathy (HCM) and 10 patients with heart failure and preserved ejection fraction (HFpEF) were studied at 1.5 Tesla. LA longitudinal strain and SR parameters were derived from SSFP cine images using dedicated CMR-FT software (2D CPA MR, TomTec, Germany). LA performance was analyzed using 4- and 2-chamber views including LA reservoir function (total strain [?_s_], peak positive SR [SRs]), LA conduit function (passive strain [?_e_], peak early negative SR [SRe]) and LA booster pump function (active strain [?_a_], late peak negative SR [SRa]).

**Results:**

In all subjects LA strain and SR parameters could be derived from SSFP images. There was impaired LA reservoir function in HCM and HFpEF (?_s_ [%]: HCM 22.1?±?5.5, HFpEF 16.3?±?5.8, Controls 29.1?±?5.3, p?<?0.01; SRs [s^?1^]: HCM 0.9?±?0.2, HFpEF 0.8?±?0.3, Controls 1.1?±?0.2, p?<?0.05) and impaired LA conduit function as compared to healthy controls (?_e_ [%]: HCM 10.4?±?3.9, HFpEF 11.9?±?4.0, Controls 21.3?±?5.1, p?<?0.001; SRe [s^?1^]: HCM ?0.5?±?0.2, HFpEF ?0.6?±?0.1, Controls ?1.0?±?0.3, p?<?0.01). LA booster pump function was increased in HCM while decreased in HFpEF (?_a_ [%]: HCM 11.7?±?4.0, HFpEF 4.5?±?2.9, Controls 7.8?±?2.5, p?<?0.01; SRa [s^?1^]: HCM ?1.2?±?0.4, HFpEF ?0.5?±?0.2, Controls ?0.9?±?0.3, p?<?0.01). Observer variability was excellent for all strain and SR parameters on an intra- and inter-observer level as determined by Bland-Altman, coefficient of variation and intraclass correlation coefficient analyses.

**Conclusions:**

CMR-FT based atrial performance analysis reliably quantifies LA longitudinal strain and SR from standard SSFP cine images and discriminates between patients with impaired left ventricular relaxation and healthy controls. CMR-FT derived atrial deformation quantification seems a promising novel approach for the study of atrial performance and physiology in health and disease states.

## Background

Left atrial (LA) function is increasingly recognized to have an incremental role in determining prognosis and risk stratification in different states of disease ¿ especially in those that are associated with ventricular diastolic dysfunction. The principal role of the LA is to modulate left ventricular filling due to three basic functional elements [1]: 1. Reservoir function (collection of pulmonary venous return during ventricular systole); 2. Conduit function (passage of blood to the left ventricle during early diastole) and 3. Contractile booster pump function (augmentation of ventricular filling during late diastole).

Echocardiographic speckle tracking has proved to be a feasible and reproducible technique to evaluate LA longitudinal strain and strain rate (SR) [2]. At the present time, the role of Cardiovascular Magnetic Resonance (CMR) to evaluate atrial function is mainly complementary to echocardiography in specific clinical instances, e.g. in diagnostic evaluation and follow-up for patients with poor echocardiographic windows [3]. CMR feature tracking (CMR-FT) ¿ a technique analogous to echocardiographic speckle tracking ¿ represents a novel approach to assess myocardial deformation directly from standard steady-state free precession (SSFP) cine CMR images and therefore does not require additional tagging sequence acquisitions [4],[5]. CMR-FT makes use of offline tracking of tissue voxel motion allowing the evaluation of longitudinal, circumferential and radial myocardial deformation. The technique has been used to analyze left and right ventricular performance in health and disease [6]-[8]. However, the feasibility of CMR-FT for the assessment of quantitative LA function and deformation has never previously been demonstrated [9]. The aim of the present study is therefore to evaluate the feasibility and reproducibility of CMR-FT for the quantification of atrial physiology as assessed with LA strain and SR.

## Methods

The study protocol was approved by the institutional review board. 10 subjects who met the conditions for HFpEF according to current consensus statements [10] (presence of signs or symptoms of congestive heart failure, presence of preserved left ventricular (LV) systolic function and echocardiographic evidence of diastolic LV dysfunction), 10 subjects with HCM (according to genetic confirmation or wall thickness???15 mm or???13 mm in case of a family history of HCM, absence of chamber dilation, absence of other systemic or cardiac disease sufficient to justify the hypertrophy) and 10 healthy controls were recruited after written informed consent was obtained. Exclusion criteria included atrial fibrillation, claustrophobia, impaired renal function, pacemaker/defibrillator devices or other metallic implants.

### CMR

All CMR measurements were performed at 1.5 Tesla (Philips Intera, Philips Achieva, Siemens Sonata or Siemens Symphony TIM) in the supine position. LV dimensions and function were assessed with ECG-gated SSFP cine sequences during brief periods of breath-holding in the following planes: 12 to 14 equidistant short-axis planes covering entire ventricles as well as 2-chamber and 4-chamber views. Typical image parameters were: repetition time (TR) 3.3 ms, echo time (TE) 1.6 ms, matrix size 228 × 220, field of view (FOV) 270 × 260 mm, slice thickness 6¿8 mm (Philips Intera); TR 2.7 ms, TE 1.3, matrix size 208 × 184, FOV 260 × 230 mm, slice thickness 8 mm (Philips Achieva); TR 30.9 ms, TE 1.3 ms, matrix size 256 × 164, FOV 400 × 256 mm, slice thickness 6¿8 mm (Siemens Sonata); TR 45.9 ms, TE 1.3 ms, matrix size 192 × 192 mm, FOV 340 × 340, slice thickness 6 mm (Siemens Symphony TIM).

### Feature tracking

**Figure 1:**
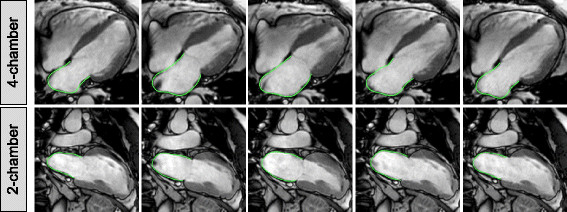
**Left atrial CMR feature tracking.** The figure shows a representative example of left atrial tracking in the 4-chamber and 2-chamber view in a patient with hypertrophic cardiomyopathy.

**Figure 2:**
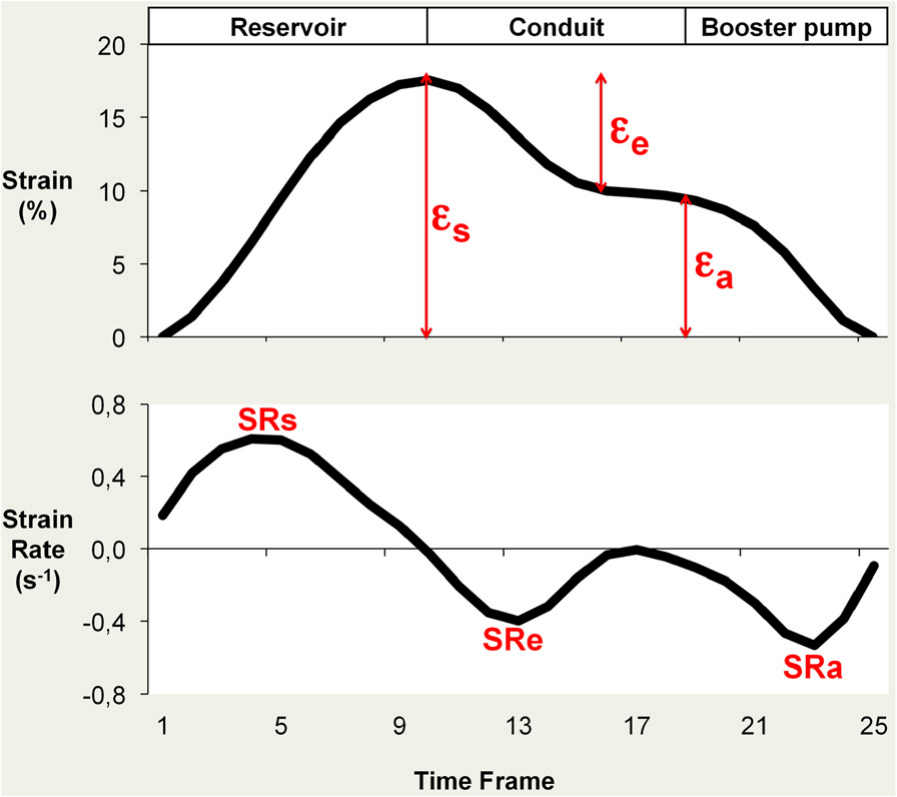
**Left atrial strain and strain rate profiles.** Left atrial function compromises reservoir, conduit and contractile booster pump function. Total strain (?_s_) and peak positive strain rate (SRs) correspond to reservoir function. Passive strain (?_e_) and peak early negative strain rate (SRe) correspond to conduit function. Active strain (?_a_) and peak late negative strain rate (SRa) correspond to contractile booster pump function.

LA myocardial feature tracking was performed using dedicated software (TomTec Imaging Systems, 2D CPA MR, Cardiac Performance Analysis, Version 1.1.2.36, Unterschleissheim, Germany). LA endocardial borders were manually traced in the 2- and 4-chamber views using a point-and-click approach when the atrium was at its minimum volume after atrial contraction. The atrial endocardial border surface was manually delineated and the automated tracking algorithm was applied. Tracking performance was visually reviewed to ensure accurate tracking of the atrial myocardium (Figure 1). In case of insufficient automated border tracking, manual adjustments were made to the initial contour and the algorithm was reapplied. The atrium was divided into six segments and averaged strain and strain rate profiles were calculated for all segments as previously described [11]. Tracking performance was visually reviewed on a segmental basis. If the tracking quality was not sufficient, e.g. due to the presence of pulmonary veins or left atrial appendage, the corresponding segment was excluded from the analysis. Tracking was repeated for three times in both the 2- and 4-chamber view. LA longitudinal strain and SR results were averaged across all three repetitions in both views. Similar to previous definitions from echocardiographic speckle tracking [11],[12] three aspects of atrial strain were analysed (Figure 2): passive strain (?_e_, corresponding to atrial conduit function), active strain (?_a,_ corresponding to atrial contractile booster pump function) and total strain, the sum of passive and active strain (?_s,_ corresponding to atrial reservoir function). Accordingly, three SR parameters were evaluated (Figure 2): peak positive strain rate (SRs, corresponding to atrial reservoir function), peak early negative strain rate (SRe, corresponding to atrial conduit function) and peak late negative strain rate (SRa, corresponding to atrial contractile booster pump function) [1],[13].

### Volumetric analysis

Volumetric analysis was performed with commercially available software (QMass, Medis, Leiden, The Netherland). Left and right ventricular ejection fraction, end-diastolic and end-systolic volumes were assessed from the short-axis stack. End-diastolic and end-systolic volumes were normalized to body surface area. Semi-automated tracings of the LA area and length were performed in the 2- and 4-chamber view. LA volumes were calculated using the previously validated biplane area-length method [14] according to the formula: LA volume (ml)?=?0.85*A_2C_*A_4C_/L, where A_2C_ and A_4C_ are the LA areas on the 2-chamber and 4-chamber views, respectively, and L is the shorter long-axis length of the LA from either the 2-chamber or the 4-chamber views. LA volumes were assessed at left ventricular end-systole (LAVmax), at left ventricular diastole just before LA contraction (LAVpre-ac) and at late left ventricular end diastole after LA contraction (LAVmin) [1],[15]. Total LA emptying fraction (LAEF Total, corresponding to atrial reservoir function), passive LA emptying fraction (LAEF passive, corresponding to atrial conduit function) and LA active emptying fraction (LAEF Booster, corresponding to atrial contractile booster pump function) were defined as fractional volume changes according to the following equations:

(1) $$\mathrm{LAEF}\;\mathrm{Total}\;=\;\frac{\left(\mathrm{LAVmax}\;¿\;\mathrm{LAVmin}\right)\;\times \;100}{\mathrm{LAVmax}}$$

(2) $$\mathrm{LAEF}\;\mathrm{Passive}\;=\;\frac{\left(\mathrm{LAVmax}\;¿\;\mathrm{LAVpre}?\mathrm{ac}\right)\;\times \;100}{\mathrm{LAVmax}}$$

(3) $$\mathrm{LAEF}\;\mathrm{Booster}\;=\;\frac{\left(\mathrm{LAVpre}?\mathrm{ac}\;¿\;\mathrm{LAVmin}\right)\;\times \;100}{\mathrm{LAVpre}?\mathrm{ac}}$$

### Statistical analysis

Statistical analysis was performed using Microsoft Excel and IBM SPSS Statistics version 22 for Macintosh. Data are expressed as mean (±standard deviation). Differences between groups in continuous variables were assessed by the Kruskal¿Wallis test. Pearson¿s correlation coefficients were calculated to investigate for potential relations between variables from CMR-FT and volumetric analyses regarding LA reservoir, conduit and contractile booster pump function. Correlation coefficients were considered weak if r???0.35, moderate if r was between 0.36-0.67 and strong if r???0.68. The intra- and inter-observer variability for strain and SR measurements were assessed by the coefficient of variation (CV), intraclass correlation coefficient (ICC) and Bland Altman analysis [16] in 10 randomly selected subjects. The CV was defined as the standard deviation of the differences divided by the mean [17]. Two independent observers analysed all cases to assess inter-observer variability (JTK & AS), while intra-observer variability was derived from the repeated analysis by the first observer (JTK) after four weeks. All statistical tests with p values?<?0.05 were considered statistically significant.

## Results

Healthy controls were younger than patients with HCM and HFpEF. LA volumes were higher in both patient groups than in healthy controls. Participant demographics are summarised in Table 1.

**Table 1:** Subject characteristics

	**HFpEF (n?=?10)**	**HCM (n?=?10)**	**Controls (n?=?10)**
**Gender (m/f)**	7/3	9/1	5/5
**Age (y)**	69.7 (58¿82)	59.1 (44¿73)	40.6 (23¿51)
**LV-EDV (ml/m**^**2**^**)**	70.6?±?12.2	71.6?±?10.7	76.9?±?12.5
**LV-ESV (ml/m**^**2**^**)**	25.8?±?5.4	23.5?±?6.4	33.4?±?7.5
**LVEF (%)**	63.6?±?3.0	67.4?±?6.4	56.9?±?4.4
**RV-EDV (ml/m**^**2**^**)**	84.3?±?11.8	71.4?±?11.2	76.6?±?14.3
**RV-ESV (ml/m**^**2**^**)**	39.0?±?13.7	24.4?±?6.1	32.1?±?8.6
**RVEF (%)**	54.7?±?8.9	65.7?±?5.7	58.5?±?4.1
Presence of MR*	2 (20%)	4 (40%)	0 (0%)
**LAV max (ml/m**^**2**^**)**	53.1?±?11.2	50.1?±?13.6	34.8?±?9.1
**LAV min (ml/m**^**2**^**)**	24.8?±?6.3	20.6?±?7.2	13.4?±?4.2
**LAV p-ac (ml/m**^**2**^**)**	40.1?±?7.9	37.7?±?12.3	23.0?±?7.7

Continuous variables are expressed as mean?±?standard deviation; age is expressed as median with range; HFpEF, heart failure with preserved ejection fraction; HCM, hypertrophic cardiomyopathy; LV, left ventricle; EDV: end-diastolic volume; ESV, end-systolic volume; EF, ejection fraction; m, male; f, female; y, years; LAV max, maximum left atrial volume; LAV min, minimum left atrial volume; LAV p-ac, left atrial volume prior to atrial contraction.

*Mitral regurgitation (MR) as defined by visual appearance of a regurgitation jet on cine SSFP.

### Feasibility of left atrial CMR-FT

LA CMR-FT was successfully performed in all subjects. Atrial strain profiles followed atrial physiology and allowed an evaluation of atrial total, passive and active strain. Furthermore, peak positive SR, peak early negative SR and peak late negative SR were successfully analysed in all cases. Tracking quality was sufficient in 321/360 segments (89.2%). In 14/30 subjects, averaged strain and SR profiles were calculated from all six segments in both the 2-chamber and 4-chamber view. In 16/30 subjects, 39 individual segments were excluded (15 segments in 4-chamber view, 24 segments in 2-chamber view) from the analyses resulting in a total proportion of 10.8% of excluded segments. Exclusion of segments was predominantly associated with poor tracking quality related to the insertion of the pulmonary veins.

Bivariate correlation demonstrated a strong to moderate relation between volumetric indexes and deformation parameters from CMR-FT for atrial reservoir, conduit and contractile booster pump functions (Table 2).

**Table 2:** Bivariate correlation of left atrial functional indexes from volumetric analysis and corresponding strain and strain rate parameters from CMR-FT

**Left atrial function**	**CMR-FT**	**Volumetric index**	**Correlation coefficient**	**P value**
**Reservoir**	?_s_	LAEF Total	0.81	< 0.001
	SRs	LAEF Total	0.73	< 0.001
**Conduit**	?_e_	LAEF Passive	0.76	< 0.001
	SRe	LAEF Passive	?0.82	< 0.001
**Booster pump**	?_a_	LAEF Booster	0.52	< 0.005
	SRa	LAEF Booster	?0.63	< 0.001

CMR-FT, Cardiovascular Magnetic Resonance myocardial feature tracking; ?, strain; SR, strain rate; LAEF, left atrial emptying fraction.

### Left atrial function in patients and controls

LA strain and SR parameters from CMR-FT revealed significantly different values between patients with HCM or HFpEF and healthy controls (Table 3). ?_s_ and SRs (both corresponding to LA reservoir function) as well as ?_e_ and SRe (both corresponding to LA conduit function) were lower in both patient groups. Interestingly, ?_a_ and SRa (both corresponding to LA booster pump function) were increased in patients with HCM while decreased in patients with HFpEF. These parameters were not significantly different in patients with mitral regurgitation as compared to patient without mitral regurgitation both in the HCM and HFpEF group.

**Table 3:** Comparison of left atrial volumetric indexes, strain (?) and strain rate (SR) parameters among patients with heart failure with preserved ejection fraction (HFpEF), patients with hypertrophic cardiomyopathy (HCM) and healthy controls

					**P value**			
		**HFpEF (n?=?10)**	**HCM (n?=?10)**	**Controls (n?=?10)**	**Overall**	**HCM vs. controls**	**HCM vs. HFpEF**	**HFpEF vs. control**
**Left atrial function**	**Left atrial volumetric index (%)**							
Reservoir	LAEF Total	53.3 (6.7)	59.4 (5.8)	61.4 (6.0)	**0.028**	0.290	0.070	**0.013**
Conduit	LAEF Passive	24.4 (4.3)	25.6 (6.2)	35.2 (8.8)	**0.010**	**0.019**	0.705	**0.005**
Booster pump	LAEF Booster	29.0 (7.3)	33.8 (5.7)	26.2 (6.8)	**0.041**	**0.013**	0.122	0.406
	**Left atrial strain (%)**							
Reservoir	?_s_	16.3 (5.8)	22.1 (5.5)	29.1 (5.3)	**0.001**	**0.008**	**0.034**	**0.001**
Conduit	?_e_	11.9 (4.0)	10.4 (3.9)	21.3 (5.1)	**<0.001**	**<0.001**	0.650	**0.001**
Booster pump	?_a_	4.5 (2.9)	11.7 (4.0)	7.8 (2.5)	**0.001**	**0.023**	**0.001**	**0.019**
	**Left atrial strain rate (s**^**?1**^**)**							
Reservoir	SRs	0.8 (0.3)	0.9 (0.2)	1.1 (0.2)	**0.023**	**0.028**	0.384	**0.017**
Conduit	SRe	?0.6 (0.1)	?0.5 (0.2)	?1.0 (0.3)	**0.002**	**0.002**	0.733	**0.003**
Booster pump	SRa	?0.5 (0.2)	?1.2 (0.4)	?0.9 (0.3)	**0.004**	0.131	**0.004**	**0.010**
	**Left atrial volume (ml/m**^**2**^**)**							
	LAV max	53.1 (11.2)	50.1 (13.6)	34.8 (9.1)	**0.004**	**0.013**	0.623	**0.002**
	LAV min	24.8 (6.3)	20.6 (7.2)	13.4 (4.2)	**0.002**	**0.021**	0.186	**0.001**
	LAV p-ac	40.1 (7.9)	37.7 (12.3)	23.0 (7.7)	**0.002**	**0.011**	0.597	**0.001**

HFpEF, heart failure with preserved ejection fraction; HCM, hypertrophic cardiomyopathy; LAEF, left atrial emptying fraction; ?, strain; SR, strain rate; LAV max, maximum left atrial volume; LAV min, minimum left atrial volume; LAV p-ac, left atrial volume prior to atrial contraction. Bold p values indicate a significance level < 0.05.

### Intra- and inter-observer variability

**Figure 3:**
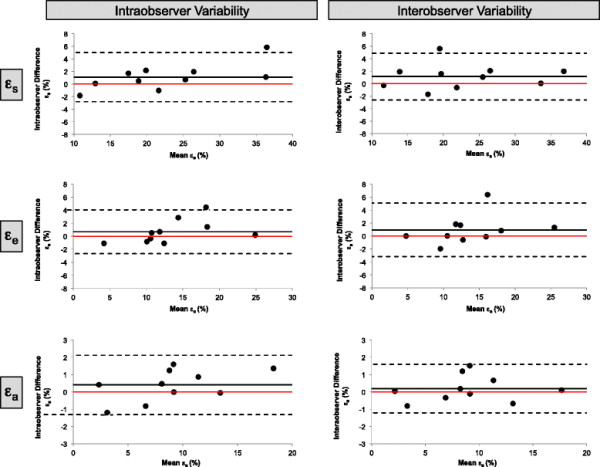
**Bland Altman Plots for intra- and inter-observer variability.** Bland Altman Plots for intra- and inter-observer variability obtained for total strain (?_s_), passive strain (?_e_) and active strain (?_a_).

**Figure 4:**
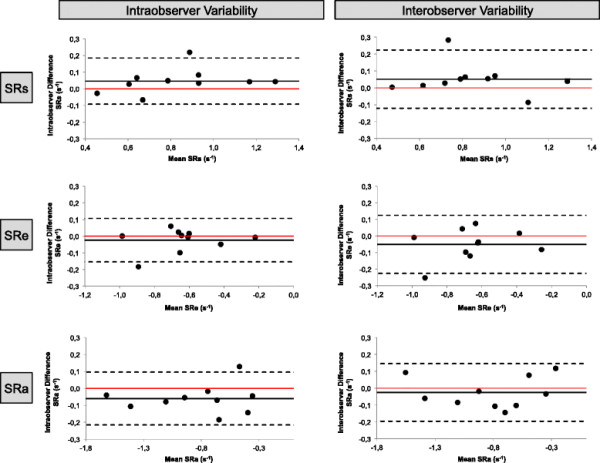
**Bland Altman Plots for intra- and inter-observer variability.** Bland Altman Plots for intra- and inter-observer variability obtained for peak positive SR (SRs), peak early negative SR (SRe) and peak late negative SR (SRa).

LA strain and SR parameters were reproducible on an intra- and inter-observer level. Bland-Altman Plots for strain and SR measurements are displayed in Figures 3 and 4, respectively. Table 4 shows ICC and CV for repeated measurements within the single and between observers.

**Table 4:** **Intra-observer and inter-observer reproducibility for strain (**?**) and strain rate (SR) parameters**

	**Intra-observer**			**Inter-observer**		
	Mean difference?±?SD*	**CV [%]**	**ICC (95% CI)**	**Mean difference?±?SD [%]**	**CV [%]**	**ICC (95% CI)**
**?**_**s**_	1.09?±?1.99	8.79	0.98 (0.93-1.00)	1.14?±?1.92	8.47	0.98 (0.92-1.00)
**?**_**e**_	0.69?±?1.71	12.59	0.97 (0.90-0.99)	0.94?±?2.10	15.34	0.96 (0.84-0.99)
**?**_**a**_	0.40?±?0.87	9.66	0.99 (0.96-1.00)	0.19?±?0.71	8.00	0.99 (0.98-1.00)
**SRs**	0.05?±?0.07	8.46	0.97 (0.88-0.99)	0.05?±?0.09	10.49	0.96 (0.82-0.99)
**SRe**	?0.02?±?0.07	10.41	0.97 (0.90-0.99)	?0.05?±?0.09	13.68	0.95 (0.78-0.99)
**SRa**	?0.06?±?0.08	9.48	0.99 (0.93-1.00)	?0.03?±?0.09	10.64	0.99 (0.96-1.00)

SD, standard deviation; CV, coefficient of variation; ICC, intraclass correlation coefficient; ?_,_ strain; SR, strain rate;

*Mean difference?±?SD are given as [%] for ? and as [s^?1^] for SR parameters.

## Discussion

Our data demonstrate that LA longitudinal strain and SR parameters can be derived from CMR-FT using routinely acquired SSFP sequences in healthy volunteers, patients with HFpEF and patients with HCM. We found a good correlation between CMR-FT derived strain and SR parameters and LA volumetric indexes regarding LA reservoir, conduit and contractile booster pump function.

CMR-FT of the LA myocardium was successfully performed in all study subjects with a sufficient tracking quality in the vast majority of segments (89.2%). Importantly the achieved tracking quality of CMR-FT is superior to the reported tracking quality of echocardiographic speckle tracking. Vianna-Pinton et al. [18] reported an exclusion rate of 6% of all participants in a group of healthy volunteers in their study using echocardiographic speckle tracking based atrial performance analysis. In the remaining study cohort, speckle tracking quality was sufficient in 91% of all segments. The technique used in this work represents the current echocardiographic standard for LA speckle tracking analysis as recommended in current guidelines [19].

The current reference standard for quantitative wall motion assessment with CMR is myocardial tagging. Myocardial tagging based strain assessment remains time-consuming since additional sequences and complex post processing are required. In the context of LA function assessment, the main problem with myocardial tagging is its limitation to ventricular deformation quantification [20]. The LA myocardium appears to be not thick enough to be labeled by grid lines. Displacement encoding with stimulated echoes (DENSE) represents a further technique that allows for quantitative assessment of myocardial deformation. Again, DENSE has not proved to be feasible in the assessment of atrial strain and SR. However, DENSE might contain a potential to assess deformation of narrow anatomical structures (e.g. the LA myocardium) since recent data indicate the feasibility to quantify aortic wall stretch [21]. In essence, CMR-FT represents the only CMR based technique that allows for LA strain and SR imaging at the present time.

### Left atrial function in patients with HCM and HFpEF

Abnormal LA function is present in patients with ventricular diastolic dysfunction. For instance, Kaminski et al. demonstrated that impaired LA contractile function was the strongest predictor of major adverse cardiac events and all-cause mortality in patients with chronic hypertension but no other prevalent cardiovascular disease [22]. Since diastolic dysfunction is present in HFpEF and HCM as well [23],[24] it is particularly interesting to investigate LA deformation in both patient groups. Looking at our data, CMR-FT revealed significantly decreased passive strain and peak early negative SR in patients with HCM and HFpEF. Both parameters correspond to atrial conduit function. LA conduit function is closely related to left ventricular compliance [1]. Therefore, decreased passive strain and decreased peak early negative SR from CMR-FT might be directly correlated with impaired ventricular relaxation. Furthermore, total strain and peak positive SR correspond to atrial reservoir function. Both parameters were decreased in HCM and HFpEF, which might refer to impaired atrial compliance. Increases in booster pump function in HCM have previously been reported [1],[25],[26]. In contrast, impaired atrial contractility has been described in HFpEF by echocardiographic speckle tracking [27]. Consistently, our results show increased active strain and peak late negative SR (both corresponding to contractile booster pump function) in HCM while decreased in HFpEF. The presence of mitral regurgitation did not seem to be associated with the increased booster pump function in the HCM group. It might be interesting to speculate whether or not the increased booster pump function with decreased reservoir and conduit function in HCM represents a form of compensated milder functional diastolic dysfunction as opposed to severe diastolic dysfunction in HFpEF with complete atrial performance impairment. Indeed Murata et al. reported initial increase in LA booster pump function at early stages of impaired left ventricular relaxation, followed by progressive decompensation of global LA performance with worsening of diastolic dysfunction at later disease stages [28]. Future studies will need to investigate whether atrial performance analysis could be a useful parameter for the identification of patients at risk for congestive heart failure with associated poor prognosis that may specifically benefit from early therapeutic interventions.

### Reproducibility

The reported amount of reproducibility and repeatability of ventricular strain and SR measurements from CMR-FT varies between studies with most studies reporting reasonable reproducibility of global strain and SR levels [5],[29]-[31]. Therefore, intra- and inter-observer variability of LA deformation indexes need to be taken into special consideration. In order to further maximise reproducibility all measurements were repeated three times in both the 2-chamber and 4-chamber view. All strain and SR parameters had excellent reproducibility both on the intra-observer as well as inter-observer level. The calculation of global strain and SR parameters (average of 6 segments) might explain the excellent reproducibility as opposed to previously reported bad segmental reproducibility of some of the ventricular CMR-FT measurements [32]. Furthermore the good reproducibility might also be associated with the good image quality of the CMR images with reliable delineation of the thin LA myocardium in contrast to LA echocardiographic speckle tracking that suffer from the far-field location of the LA and reduced signal-to-noise ratio. On the other side, lower temporal resolution of CMR images might affect deformation analysis, e.g. the capacity to measure peak strain rates. However a reasonable inter-modality agreement for left ventricular longitudinal strain analysis has been demonstrated between CMR-FT and echocardiographic speckle tracking despite differences in temporal resolution [33]. Future studies will need to investigate the effect of these parameters for LA strain and SR imaging with CMR-FT.

### Limitations

We have performed a feasibility study to demonstrate the use of CMR-FT for the assessment of atrial deformation. The sample size of healthy volunteers and patients was relatively small and has not been powered to detect gross differences between groups. Nevertheless we were able to detect significant differences between health and disease and future studies will need to investigate the clinical utility of CMR-FT derived LA strain and SR parameters in a larger cohort of patients with different pathologies. CMR measurements were performed on different scanner types. Importantly the reproducibility assessed in the current study was excellent for all parameters irrespective of scanner type. This is in line with previous studies demonstrating similar reproducibility of CMR-FT ventricular strain analysis at 1.5 and 3 Tesla [30],[31].

Furthermore LA CMR-FT faces several challenges as opposed to ventricular deformation tracking that may limit the performance of the LA tracking: 1. The LA myocardial wall is thinner than the ventricular myocardial wall, 2. LA anatomy is more variable than ventricular anatomy, 3. LA physiology is quite complex (reservoir function, conduit function, booster pump function), 4. The presence of pulmonary veins and left atrial appendage can compromise tracking quality. Notwithstanding these facts, CMR-FT analysis was successful in all controls and patients. Tracking quality was sufficient in the vast majority of segments (89.2%) and reproducibility was excellent.

## Conclusions

CMR-FT allows derivation of LA longitudinal deformation mechanics directly from standard SSFP cine images. LA strain and SR parameters were highly reproducible on an intra- and inter-observer basis. Differences in strain and SR parameters between healthy controls and patients with HCM or HFpEF were in consistence with published literature. LA CMR myocardial deformation analysis with feature tracking may have potential clinical and research applications. Further studies in larger cohorts should follow to establish the clinical utility and the prognostic implications of this technique.

## Abbreviations

CMR: Cardiovascular Magnetic Resonance

CMR-FT: CMR feature tracking

CV: Coefficient of variation

?: Strain

FOV: Field of view

HCM: Hypertrophic cardiomyopathy

HFpEF: Heart failure with preserved ejection fraction

ICC: Intraclass correlation coefficient

LA: Left atrial

LAEF: Left atrial emptying fraction

LAV: Left atrial volume

SR: Strain rate

SSFP: Steady-state free precession

TE: Echo time

TR: Repetition time

## Competing interests

The authors declare that they have no competing interests.

## Authors¿ contributions

JTK and AS designed the study protocol, performed data acquisition and measurements, performed statistical analysis and drafted the manuscript. FE, GH, JL revised the manuscript, participated in the scientific discussion during the study. SK, AC, AV, MS, JMS, WS, NB, CUB revised the manuscript and performed data acquisition. All authors read and approved the final manuscript.

## References

[B1] Hoit BD Left atrial size and function: role in prognosis J Am Coll Cardiol 2014 63 493 505 10.1016/j.jacc.2013.10.055 24291276

[B2] Vieira MJ Teixeira R Goncalves L Gersh BJ Left atrial mechanics: echocardiographic assessment and clinical implications J Am Soc Echocardiogr 2014 27 463 78 10.1016/j.echo.2014.01.021 24656882

[B3] To AC Flamm SD Marwick TH Klein AL Clinical utility of multimodality LA imaging: assessment of size, function, and structure JACC Cardiovasc Imaging 2011 4 788 98 10.1016/j.jcmg.2011.02.018 21757171

[B4] Kowallick JT Edelmann F Lotz J Lamata P Schuster A Imaging diastolic dysfunction with cardiovascular magnetic resonance J Cardiol Ther 2014 1 58 64

[B5] Schuster A Kutty S Padiyath A Parish V Gribben P Danford DA Makowski MR Bigalke B Beerbaum P Nagel E Cardiovascular magnetic resonance myocardial feature tracking detects quantitative wall motion during dobutamine stress J Cardiovasc Magn Reson 2011 13 58 10.1186/1532-429X-13-58 21992220 PMC3217847

[B6] Morton G Schuster A Jogiya R Kutty S Beerbaum P Nagel E Inter-study reproducibility of cardiovascular magnetic resonance myocardial feature tracking J Cardiovasc Magn Reson 2012 14 43 10.1186/1532-429X-14-43 22721175 PMC3461471

[B7] Onishi T Saha SK Ludwig DR Marek JJ Cavalcante JL Schelbert EB Schwartzman D Gorcsan J 3rd Feature tracking measurement of dyssynchrony from cardiovascular magnetic resonance cine acquisitions: comparison with echocardiographic speckle tracking J Cardiovasc Magn Reson 2013 15 95 10.1186/1532-429X-15-95 24134158 PMC4016574

[B8] Schuster A Paul M Bettencourt N Morton G Chiribiri A Ishida M Hussain S Jogiya R Kutty S Bigalke B Perera D Nagel E Cardiovascular magnetic resonance myocardial feature tracking for quantitative viability assessment in ischemic cardiomyopathy Int J Cardiol 2013 166 413 20 10.1016/j.ijcard.2011.10.137 22130224

[B9] Hoit BD Reply to letter to the editor: feature tracking cardiac magnetic resonance imaging in the assessment of left atrial function J Am Coll Cardiol 2014 63 2435 36 10.1016/j.jacc.2014.02.544 24632275

[B10] Paulus WJ Tschope C Sanderson JE Rusconi C Flachskampf FA Rademakers FE Marino P Smiseth OA De Keulenaer G Leite-Moreira AF Borbély A Edes I Handoko ML Heymans S Pezzali N Pieske B Dickstein K Fraser AG Brutsaert DL How to diagnose diastolic heart failure: a consensus statement on the diagnosis of heart failure with normal left ventricular ejection fraction by the Heart Failure and Echocardiography Associations of the European Society of Cardiology Eur Heart J 2007 28 2539 50 10.1093/eurheartj/ehm037 17428822

[B11] Ring L Rana BS Wells FC Kydd AC Dutka DP Atrial function as a guide to timing of intervention in mitral valve prolapse with mitral regurgitation JACC Cardiovasc Imaging 2014 7 225 32 10.1016/j.jcmg.2013.12.009 24529886

[B12] Cameli M Caputo M Mondillo S Ballo P Palmerini E Lisi M Marino E Galderisi M Feasibility and reference values of left atrial longitudinal strain imaging by two-dimensional speckle tracking Cardiovasc Ultrasound 2009 7 6 10.1186/1476-7120-7-6 19200402 PMC2652427

[B13] Kutty S Padiyath A Li L Peng Q Rangamani S Schuster A Danford DA Functional maturation of left and right atrial systolic and diastolic performance in infants, children, and adolescents J Am Soc Echocardiogr 2013 26 398 409 e392 10.1016/j.echo.2012.12.016 23337737

[B14] Sievers B Kirchberg S Addo M Bakan A Brandts B Trappe HJ Assessment of left atrial volumes in sinus rhythm and atrial fibrillation using the biplane area-length method and cardiovascular magnetic resonance imaging with TrueFISP J Cardiovasc Magn Reson 2004 6 855 63 10.1081/JCMR-200036170 15646889

[B15] Jarvinen V Kupari M Hekali P Poutanen VP Assessment of left atrial volumes and phasic function using cine magnetic resonance imaging in normal subjects Am J Cardiol 1994 73 1135 38 10.1016/0002-9149(94)90298-4 8198044

[B16] Bland JM Altman DG Statistical methods for assessing agreement between two methods of clinical measurement Lancet 1986 1 307 10 10.1016/S0140-6736(86)90837-8 2868172

[B17] Grothues F Smith GC Moon JC Bellenger NG Collins P Klein HU Pennell DJ Comparison of interstudy reproducibility of cardiovascular magnetic resonance with two-dimensional echocardiography in normal subjects and in patients with heart failure or left ventricular hypertrophy Am J Cardiol 2002 90 29 34 10.1016/S0002-9149(02)02381-0 12088775

[B18] Vianna-Pinton R Moreno CA Baxter CM Lee KS Tsang TS Appleton CP Two-dimensional speckle-tracking echocardiography of the left atrium: feasibility and regional contraction and relaxation differences in normal subjects J Am Soc Echocardiogr 2009 22 299 305 10.1016/j.echo.2008.12.017 19258177

[B19] Mor-Avi V Lang RM Badano LP Belohlavek M Cardim NM Derumeaux G Galderisi M Marwick T Nagueh SF Sengupta PP Sicari R Smiseth OA Smulevitz B Takeuchi M Thomas JD Vannan M Voigt JU Zamorano JL Current and evolving echocardiographic techniques for the quantitative evaluation of cardiac mechanics: ASE/EAE consensus statement on methodology and indications endorsed by the Japanese Society of Echocardiography J Am Soc Echocardiogr 2011 24 277 313 10.1016/j.echo.2011.01.015 21338865

[B20] Attili AK Schuster A Nagel E Reiber JH van der Geest RJ Quantification in cardiac MRI: advances in image acquisition and processing Int J Cardiovasc Imaging 2010 26 Suppl 1 27 40 10.1007/s10554-009-9571-x 20058082 PMC2816803

[B21] Haraldsson H Hope M Acevedo-Bolton G Tseng E Zhong X Epstein FH Ge L Saloner D Feasibility of asymmetric stretch assessment in the ascending aortic wall with DENSE cardiovascular magnetic resonance J Cardiovasc Magn Reson 2014 16 6 10.1186/1532-429X-16-6 24400865 PMC3895850

[B22] Kaminski M Steel K Jerosch-Herold M Khin M Tsang S Hauser T Kwong RY Strong cardiovascular prognostic implication of quantitative left atrial contractile function assessed by cardiac magnetic resonance imaging in patients with chronic hypertension J Cardiovasc Magn Reson 2011 13 42 10.1186/1532-429X-13-42 21843343 PMC3195715

[B23] Kurt M Wang J Torre-Amione G Nagueh SF Left atrial function in diastolic heart failure Circ Cardiovasc Imaging 2009 2 10 5 10.1161/CIRCIMAGING.108.813071 19808559

[B24] D'Andrea A De Corato G Scarafile R Romano S Reigler L Mita C Allocca F Limongelli G Gigantino G Liccardo B Cuomo S Tagliamonte G Caso P Calbrò R Left atrial myocardial function in either physiological or pathological left ventricular hypertrophy: a two-dimensional speckle strain study Br J Sports Med 2008 42 696 702 10.1136/bjsm.2007.041210 18070810

[B25] Anwar AM Soliman OI Geleijnse ML Michels M Vletter WB Nemes A ten Cate FJ Assessment of left atrial ejection force in hypertrophic cardiomyopathy using real-time three-dimensional echocardiography J Am Soc Echocardiogr 2007 20 744 48 10.1016/j.echo.2006.11.017 17543746

[B26] Anwar AM Soliman OI Nemes A Geleijnse ML ten Cate FJ An integrated approach to determine left atrial volume, mass and function in hypertrophic cardiomyopathy by two-dimensional echocardiography Int J Cardiovasc Imaging 2008 24 45 52 10.1007/s10554-007-9224-x 17541727 PMC2121119

[B27] Morris DA Gailani M Vaz Perez A Blaschke F Dietz R Haverkamp W Ozcelik C Left atrial systolic and diastolic dysfunction in heart failure with normal left ventricular ejection fraction J Am Soc Echocardiogr 2011 24 651 62 10.1016/j.echo.2011.02.004 21458230

[B28] Murata M Iwanaga S Tamura Y Kondo M Kouyama K Ogawa S A real-time three-dimensional echocardiographic quantitative analysis of left atrial function in left ventricular diastolic dysfunction Am J Cardiol 2008 102 1097 102 10.1016/j.amjcard.2008.05.067 18929716

[B29] Hor KN Gottliebson WM Carson C Wash E Cnota J Fleck R Wansapura J Klimeczek P Al-Khalidi HR Chung ES Benson DW Mazur W Comparison of magnetic resonance feature tracking for strain calculation with harmonic phase imaging analysis JACC Cardiovasc Imaging 2010 3 144 51 10.1016/j.jcmg.2009.11.006 20159640

[B30] Schuster A Morton G Hussain ST Jogiya R Kutty S Asrress KN Makowski MR Bigalke B Perera D Beerbaum P Nagel E The intra-observer reproducibility of cardiovascular magnetic resonance myocardial feature tracking strain assessment is independent of field strength Eur J Radiol 2013 82 296 301 10.1016/j.ejrad.2012.11.012 23246014

[B31] Singh A, Steadman CD, Khan JN, Horsfield MA, Bekele S, Nazir SA, Kanagala P, Masca NG, Clarysse P, McCann GP. **Intertechnique agreement and interstudy reproducibility of strain and diastolic strain rate at 1.5 and 3 tesla: a comparison of feature-tracking and tagging in patients with aortic stenosis.** *J Magn Reson Imaging.* 2014. doi:10.1002/jmri.24625. 10.1002/jmri.24625 24700404

[B32] Wu L Germans T Guclu A Heymans MW Allaart CP van Rossum AC Feature tracking compared with tissue tagging measurements of segmental strain by cardiovascular magnetic resonance J Cardiovasc Magn Reson 2014 16 10 10.1186/1532-429X-16-10 24450803 PMC3926943

[B33] Padiyath A Gribben P Abraham JR Li L Rangamani S Schuster A Danford DA Pedrizzetti G Kutty S Echocardiography and cardiac magnetic resonance-based feature tracking in the assessment of myocardial mechanics in tetralogy of Fallot: an intermodality comparison Echocardiography 2013 30 203 10 10.1111/echo.12016 23167248

